# Predicting Depression in Dementia Caregivers: Do Religious/Spiritual Struggles Play a Role?

**DOI:** 10.1093/geroni/igaa057.3186

**Published:** 2020-12-16

**Authors:** Elizabeth MacDougall

**Affiliations:** Bridgewater College, Bridgewater, Virginia, United States

## Abstract

Using a stress process framework model, this study is the first to comprehensively examine the role that religious/spiritual struggles play in the lives of informal dementia caregivers. A convenience sample of 156 informal dementia caregivers completed a scale measuring six domains of religious/spiritual struggles, as well as other measures of primary stressors, background/contextual variables, and mental health outcome (depression). Overall levels of religious/spiritual struggle were low, but 26 percent of the sample were classified as possible cases of clinically significant religious/spiritual struggle for at least one of the six domains. Of this group, 49 percent acknowledged struggles with ultimate meaning. Religious/spiritual struggles predicted greater self-reported depression over and above number of care recipient problem behaviors (primary stressor), caregiver sex, and caregiver personality (i.e., emotional stability). Although no individual domain of religious/spiritual struggle emerged as most salient, caregivers reported significantly more ultimate meaning struggles than demonic or interpersonal struggles. These findings support the growing body of research suggesting that religious/spiritual struggles serve as a secondary stressor, adding predictive power to background/contextual factors and to primary stressors for informal dementia caregiver mental health outcomes. Further research in this area may advance efforts to better equip both secular and religious professionals to provide evidence-based counsel to informal dementia caregivers.

